# Protease-catalyzed enzymatic synthesis of antifreeze peptide oligomers and their cryopreservation performance

**DOI:** 10.1016/j.isci.2025.113802

**Published:** 2025-10-24

**Authors:** Xiaocheng Pan, Qi Wu, Bo Xia

**Affiliations:** 1 Department of Biological Environment, Jiyang College of Zhejiang A&F University, Zhuji 311800, China; 2 Department of Chemistry, Zhejiang University, Hangzhou 310080, China

**Keywords:** Analytical chemistry, Catalysis, Enzyme engineering, Chemical process

## Abstract

Sub-zero preservation is essential in biomedical fields like tissue engineering, gene therapy, and transplantation. Traditional cryoprotectants like dimethyl sulfoxide are toxic and can alter cell epigenetics, necessitating safer alternatives. Herein, an enzymatic synthesis of antifreeze peptide oligomers (AFPOs) using protease-catalyzed polymerization is introduced. Employing proteases as catalysts, AFPOs were synthesized by condensing *L*-alanyl-alanyl-threonine (*L*-AAT) as a model monomer, and this method was extended to produce two additional sequences, poly(*L*-alanyl-threonine-alanyl) (*L*-PATA) and poly(*L*-threonine-alanyl-alanyl) (*L*-PTAA). Notably, using *L-*alanyl-threonyl-alanine (*L*-ATA) as the monomer, a degree of polymerization up to 14 was achieved. Although identical amino acid compositions were shared by all AFPOs, measurable antifreeze activity was exhibited only by *L*-PTAA, indicating that monomer sequence—and thus detailed backbone architecture—rather than composition alone governs antifreeze performance. Excellent antifreezing properties were demonstrated by *L*-PTAA, marking it as safe, sustainable alternatives to conventional cryoprotectants. These findings highlight the effectiveness of enzymatic synthesis methods for producing functional AFPOs.

## Highlights

• A novel protease-catalyzed method to synthesize antifreeze peptide oligomers
• Synthetic L-PTAA peptide oligomers exhibited significant THA and IRI capability
• Only L-PTAA showed antifreeze activity despite identical amino acid compositions
• Successful cryopreservation of sheep red blood cells using L-PTAA as cryoprotectant

## Graphical abstract

**Figure:**
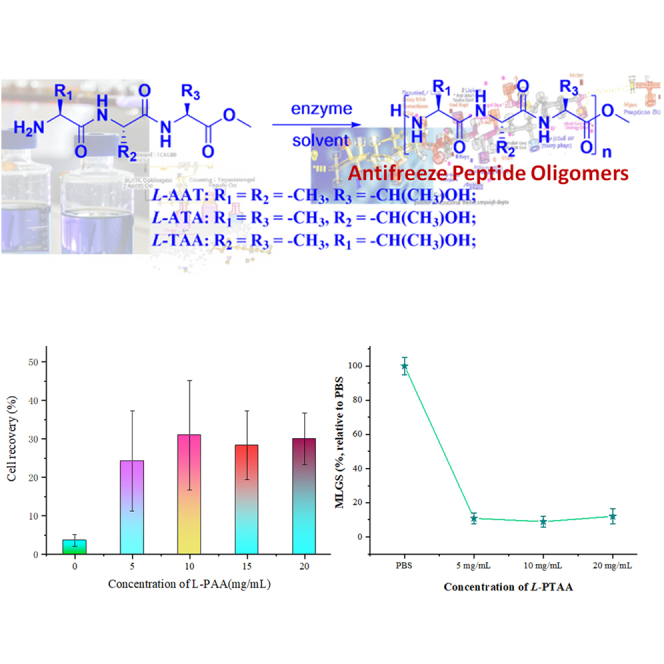

## Introduction

The sub-zero storage of biological materials is crucial in diverse fields such as tissue engineering, gene therapy, therapeutic protein production, and transplantation.^1^ However, conventional cryoprotectants like dimethyl sulfoxide (DMSO) are inherently toxic and can disrupt the epigenetic profiles of cells.^2^ Consequently, strategies aimed at inhibiting ice formation and growth are considered more effective for enhancing sub-zero storage conditions. Antifreeze peptides (AFPs) produced by fish, insects, plants, and soil bacteria serve as potent ice recrystallization inhibitors, protecting these organisms from freezing damage.^3^^,^^4^ AFPs lower the freezing temperature of solutions without affecting their melting point and inhibit ice recrystallization, preventing cellular damage.^5^ These unique properties have led to widespread applications of AFPs in enhancing the quality of frozen foods, cryopreservation of cells, cryosurgery, and aquaculture.^6^ Therefore, investigating environmentally friendly synthesis strategies for AFPs remains of significant value.

Traditionally, solid-phase peptide synthesis (SPPS) and recombinant bacterial expression systems are the primary methods employed for synthesizing AFPs.^7^ However, both approaches have limitations. SPPS involves laborious protection-deprotection steps, requires rigorous reaction conditions, and utilizes toxic reagents, leading to complex procedures and environmental concerns. Meanwhile, recombinant bacterial expression systems often yield low amounts of product and necessitate extensive purification procedures, making the process time-consuming and labor-intensive. Enzymatic peptide synthesis has emerged as a more environmentally friendly alternative and has been explored over the past decades.^8^^,^^9^ Previously, our group reported a lipase-catalyzed synthesis of poly-aspartates.^10^ Building upon this foundation, we aim to develop an enzymatic synthesis strategy for antifreeze peptide oligomers (AFPOs).

To date, type I AFPs from polar fish feature over 70% L-alanine residues, whose unique structural characteristics are vital for antifreeze activity.^11^ Threonine (Thr), a hydrophilic amino acid, forms hydrogen bonds with ice, further enhancing ice-binding and antifreeze properties. A series of studies has underscored the specific importance of these alanine and threonine residues in antifreeze function.^12^^,^^13^^,^^14^^,^^15^^,^^16^ Moreover, polar fish antifreeze glycoproteins comprise repeating alanyl-alanyl-threonyl (Ala-Ala-Thr, AAT) tripeptide units glycosylated at the Thr residue by a disaccharide via a glycosidic bond.^17^ Inspired by this natural motif, the design and synthesis of AFPOs based on the Ala-Ala-Thr sequence have attracted considerable interest in their potential antifreeze performance. Besides, to probe the effect of amino acid sequence, we polymerized three tripeptide motifs, AAT, ATA, and TAA, into the corresponding AFPOs, denoted poly-AAT, poly-ATA, and poly-TAA, respectively. Proteases, which naturally hydrolyze proteins, can function as transferases by catalyzing the transfer of an acyl group to an amino-acid-derived nucleophile through a kinetically controlled process.^18^ The mild reaction conditions, absence of side-chain protection, and prevention of racemization render enzymatic peptide synthesis more convenient and greener.^19^ Recent studies have demonstrated that protease-catalyzed oligomerization provides a powerful and efficient approach to peptide synthesis.^20^^,^^21^ Accordingly, it was applied to prepare AFPOs based on the AAT sequence.

Herein, we demonstrate an enzymatic strategy for AFPO synthesis. Protease-catalyzed condensation of *L*-alanyl-alanyl-threonine (*L*-AAT) was employed as the model reaction, and two other AFP sequences, poly(*L*-alanyl-threonine-alanyl) (*L*-PATA) and poly(*L*-threonine-alanyl-alanyl) (*L*-PTAA), were prepared under optimal conditions. The highest degree of polymerization (DP) observed reached up to 14 when *L-*alanyl-threonyl-alanine (*L*-ATA) was used as the monomer. The antifreeze properties of these three AFPOs were determined by differential scanning calorimetry (DSC) and ice recrystallization inhibition (IRI) activity analysis. Initially, we hypothesized that all synthetic AFPOs would exhibit similar antifreeze activity, since they share backbones built from the same three amino acids despite being assembled from different monomers. In practice, however, only *L*-PTAA showed measurable antifreeze behavior. Although these polymers have identical amino acid compositions, their monomer sequences—and therefore the detailed architectures of their “backbones”—differ. These findings thus reveal that sequence order, not merely composition, is a critical determinant of antifreeze performance in AFPOs.

## Results and discussions

### Optimization of polymerization conditions

**Figure 1:**
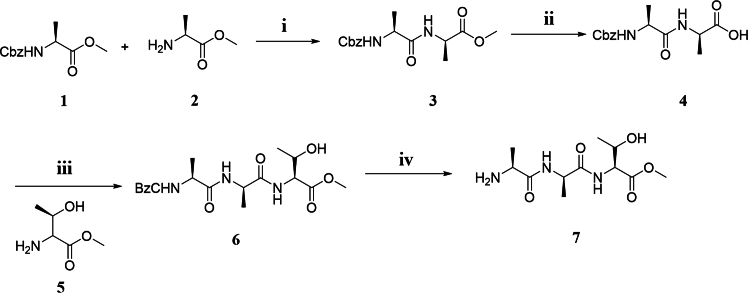
Synthesis route of monomer (i) Substrates (0.1 mol), DCC (0.11 mol), HOBt (0.12 mol), DCM, room temperature (RT), overnight. (ii) Substrates (0.01 mol), MeOH (20 mL), NaOH (2N, 10 mL, dropwise), RT, 3 h. (iii) Substrates (0.1 mol), DCC (0.11 mol), HOBt (0.12 mol), DCM, RT, overnight. (iv) 0.01 mol substrate, Pd/C (5%), 1.0 atm, overnight.

*Candida antarctica* lipase B (CALB, Novozym 435) is renowned for its versatility and has been widely applied in various organic transformations, including kinetic resolution and polymerization reactions. Its efficiency in facilitating such reactions makes it a valuable tool in synthetic chemistry.^22^^,^^23^^,^^24^^,^^25^^,^^26^^,^^27^^,^^28^^,^^29^ Previous research has successfully employed CALB in the synthesis of poly-aspartates, demonstrating its potential in catalyzing the formation of peptide bonds in polymeric structures. In this work, we aimed to utilize CALB to catalyze the polymerization of *L*-AAT (preparation was performed according to the procedure shown in Figure 1) to produce AFPOs through an enzymatic approach. To explore the potential of other enzymes in performing this function, we also investigated the catalytic activities of *Bacillus subtilis* alkaline protease (BSAP), porcine pancreas lipase (PPL), and neutral protease chosen based on their known catalytic properties and availability. The results, summarized in Table 1, revealed that only BSAP, PPL, and CALB exhibited catalytic activity toward the polymerization of *L*-AAT (Table 1, entries 1–3). Specifically, these enzymes facilitated the formation of peptide bonds between *L*-AAT monomers under the conditions tested, leading to the synthesis of oligomeric peptides. Further analysis showed that BSAP displayed superior catalytic performance compared to both CALB and PPL. The enhanced activity of BSAP may be attributed to its optimal active site configuration for accommodating the *L*-AAT substrate and facilitating peptide bond formation. Considering the outstanding catalytic efficiency of BSAP, it was selected as the preferred catalyst for further optimization studies.

**Table 1:** The polymerization of *L*-AAT catalyzed by several enzymes


Entry	Enzyme^a^	DP^b^	Yield (%)^c^
1	BSAP	6	49
2	PPL	4	32
3	Novozym 435	3	41
4	pancreatin	–d	–
5	neutral protease	–	–
6	bromelain	–	–
7	papain	2	<10
8	subtilisin	–	–
9	CAL-A	–	–

a Conditions: 500 mg L-AAT, 100 mg enzyme, 5.0 mL solvent (THF/DMF = 4:1), 50°C, 72 h.

b The DP was calculated through ^1^H-NMR analysis.

c Isolated yields.

d Polymerization did not occur.

The choice of solvent plays a critical role in enzymatic reactions, significantly influencing enzyme activity, substrate solubility, and overall reaction efficiency. Recognizing this importance, we sought to optimize the solvent system to achieve higher molecular weights of poly(*L*-alanyl-alanyl-threonine) (*L*-PAAT), which is crucial for enhancing the functional properties of the AFPOs we aim to synthesize. To this end, several organic solvent systems were systematically tested to determine their effect on the enzymatic polymerization of *L*-AAT. The results of these experiments are presented in Table 2, providing a comprehensive overview of the DP and yield obtained with each solvent system tested. Among the solvent systems evaluated, the mixed solvent of isopropanol (IPA) and *N,N*-dimethylformamide (DMF) in a 4:1 volume ratio (v/v) exhibited the best performance (Table 2, entry 3). Condensation reactions conducted in this mixed solvent resulted in a DP of^30^ and a yield of 68%, indicating a substantial improvement over other solvent system. The superior performance of the IPA/DMF (4:1) solvent system can be attributed to several factors. IPA is a protic solvent known to stabilize enzymes by maintaining their native conformation, while DMF is an aprotic polar solvent that enhances the solubility of the substrate. The synergistic effect of these two solvents likely promotes better interaction between the enzyme and substrate, leading to higher polymerization efficiency. In contrast, when DMF was used as the sole solvent (Table 2, entry 5), poor results were observed, with significantly lower DP and yield. This outcome suggests that DMF alone may adversely affect enzyme stability or may not sufficiently support the necessary interactions for effective polymerization. The absence of a protic component like IPA could lead to unfavorable conditions for the enzyme’s catalytic activity. Similarly, when IPA was replaced by ethanol in the mixed solvent system (Table 2, entry 2), comparable results were observed, but they did not surpass the performance of the IPA/DMF mixture. Based on these findings, the mixed solvent of IPA and DMF in a 4:1 volume ratio was chosen for further investigations. This solvent system appears to provide the best balance between enzyme stability and substrate solubility, creating an environment conducive to achieving higher molecular weights and yields in the enzymatic synthesis of *L*-PAAT.

**Table 2:** Effect of solvent on the polymerization

Entry	Solvent^a^	DP^b^	Yield (%)^c^
1	THF/DMF (4:1)	3	36
2	EtOH/DMF (4:1)	5	39
3	IPA/DMF (4:1)	12	68
4	TAA/DMF (4:1)	2	29
5	DMF	2–3	31
6	DMSO	–d	–
7	EA	–	–
8	acetonitrile/DMF (4/1)	2	11%
9	water	–	–

a Conditions: 500 mg AAT, 100 mg BSAP, 5.0 mL solvent, 50°C, 72 h.

b The DP were determined by ^1^H-NMR analysis.

c Isolated yields.

d Polymerization did not occur.

### Structure and molecular weight confirmation of synthetic AFPOs

**Figure 2:**
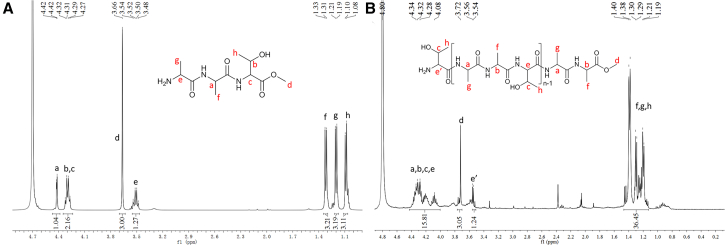
^1^H-NMR spectra of L-AAT (A) and L-PAAT (B) in D_2_O.

^1^H-NMR spectroscopy was employed to analyze both the monomer and polymer forms of *L*-AAT to confirm successful polymerization and calculate the DP of the resulting antifreeze polymer *L*-PAAT. The ^1^H-NMR spectra of the monomer L-AAT and its corresponding polymer *L*-PAAT are presented in Figure 2. Comparing these spectra provides insights into the structural changes that occur during the polymerization process. In the spectrum of the monomer *L*-AAT (Figure 2A), the peak at around 4.40–4.42 ppm (labeled as *a*) is attributed to the proton attached to the chiral carbon atom on the main chain of the second alanine residue. The peak at 3.66 ppm (labeled as *d*) corresponds to the terminal methyl ester group, and the peak at 3.48–3.54 ppm (labeled as *e*) is assigned to the proton linked to the chiral carbon atom of the first alanine residue. These three proton signals (*a*, *d*, and *e*) serve as integral benchmarks in the ^1^H-NMR spectra of both the monomer *L*-AAT and the polymer *L*-PAAT because their chemical shifts and integral areas remain unchanged during polymerization. This invariance makes them reliable reference points for calculating the degree of polymerization.

Upon polymerization, notable changes appear in the ^1^H-NMR spectrum of *L*-PAAT (Figure 2A). Specifically, new proton signals emerge at around 3.96–4.34 ppm (labeled as *b* and *c*), which are assigned to the methine protons of the internal threonine and alanine residues within the polymer chain. The appearance and increased intensity of these signals indicate the formation of new peptide bonds between *L*-AAT monomers during the polycondensation process, as these internal residues are not present in the monomer. Moreover, the proton signals corresponding to the methyl groups on the side chains of alanine (labeled as *f* and *g*) and threonine (labeled as *h*) show a significant increase in intensity after polymerization when comparing Figures 2A and 2B. This enhancement reflects the greater number of amino acid residues present in the polymer compared to the monomer, further confirming successful polymerization. By analyzing these spectral changes, we confirmed the successful synthesis of *L*-PAAT. The ^1^H-NMR spectrum of *L*-ATA, *L*-TAA, *L*-PATA, and *L*-PTAA was listed in Figures S1 and S5. Both ^1^H-NMR indicated the successful synthesis of corresponding AFPOs.

**Figure 3:**
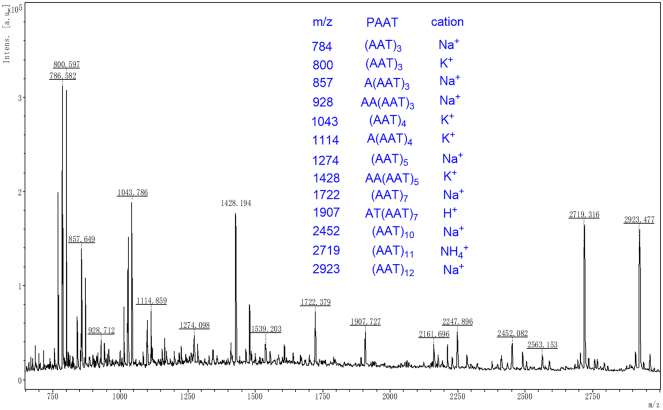
MALDI-TOF mass spectrum of L-PAAT

MALDI-TOF mass spectrometry is a highly sensitive analytical technique widely used for analyzing biomolecules, such as peptides and proteins, due to its ability to accurately measure the mass-to-charge ratios (m/z) of ions and to handle complex mixtures without extensive purification. Thus, it was utilized to determine the DP and molecular weight distribution of the synthetic peptides. The MALDI-TOF mass spectrum of *L*-PAAT is presented in Figure 3. In the mass spectrum, most of the observed peaks correspond to peptide ions formed by the addition of potassium (K^+^), proton (H^+^), ammonium ion (NH^4+^), and sodium (Na^+^) cations. This is a common occurrence in MALDI-TOF analyses, as these cations are present in the sample, matrix, or instrument and readily form adducts with peptides and proteins during ionization. The mass spectrum exhibits a series of repeating peaks spaced 243 m/z units apart. This mass difference corresponds precisely to the molar mass of an *L*-AAT monomer unit, indicating that each successive peak represents a peptide chain lengthened by one additional *L*-AAT unit. The consistent spacing of these peaks confirms the stepwise addition of monomer units during the polymerization process, reflecting the successful formation of peptide bonds between L-AAT monomers and the growth of the peptide chain. The largest mass observed in the spectrum was 2,923 m/z (addition of Na^+^), which corresponds to *L*-PAAT with a DP of 12. This suggests that the polymer chain consists of 12 repeating units of *L*-AAT, resulting in a peptide composed of 36 amino acid residues (since each *L*-AAT unit contains three amino acids: two alanine and one threonine). The detection of such high-molecular-weight species indicates that the enzymatic polymerization efficiently produces longer peptide chains, which is advantageous for enhancing the antifreeze properties of the peptides. The strongest signal in the mass spectrum was observed at 784 m/z, corresponding to *L*-PAAT with a DP of 3. This peak represents a peptide chain containing three *L*-AAT units and thus nine amino acid residues. The prominence of this peak suggests that shorter polymer chains are either more abundant in the sample or are more efficiently ionized and detected under the MALDI-TOF conditions used. It is important to note that MALDI-TOF signal intensities are not directly proportional to the concentration of the analyte species due to factors such as ionization efficiency, desorption efficiency, and the detection bias toward certain molecular weights. Therefore, while the mass spectrum provides valuable qualitative information about the range of polymer chain lengths present, it does not allow for the quantitative determination of the total product yield or the precise distribution of chain lengths.

Additionally, the MALDI-TOF mass spectra of *L*-PATA and *L*-PTAA were recorded and are shown in Figures S6 and S7, respectively. For *L*-PATA, the largest mass observed was 3,431 m/z, corresponding to a DP of 14. This indicates that the polymerization of *L*-PATA proceeds effectively, producing long peptide chains that are even longer than those observed for *L*-PAAT. Similarly, for *L*-PTAA, the largest mass observed was 2,719 m/z, also corresponding to a DP of 11. Overall, the MALDI-TOF analysis provides valuable insights into the molecular weight distribution and chain lengths of the synthesized peptides. The data confirm that enzymatic polymerization yields peptides with varying degrees of polymerization, ranging from short oligomers to longer chains with DPs from 11 to 14. By expanding on the MALDI-TOF results, we gain a deeper understanding of the polymerization process, the molecular weight distribution, and the capability of the enzymatic method to produce peptides with substantial chain lengths. This detailed analysis underscores the importance of characterizing the synthesized peptides to assess their potential effectiveness as antifreeze agents and to guide the optimization of polymerization conditions for desired properties.

### Thermal properties of synthetic peptides

**Figure 4:**
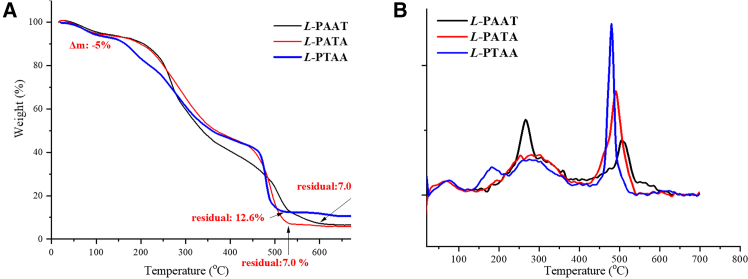
Thermal properties of synthetic oligopeptides (A) TGA traces of synthetic AFOPs. (B) The first derivative curves of TGA traces.

To evaluate the thermal stability of the synthesized peptides, thermogravimetric analysis (TGA) was performed. The TGA curves of the peptides are presented in Figure 4. The results reveal distinct thermal decomposition patterns for each peptide, providing insights into their stability and decomposition mechanisms. Generally, decomposition occurs in two main steps for all peptides except *L*-PTAA, which decomposes in three distinct steps (see Figure 4B). All three synthetic AFPOs exhibit an initial weight loss of approximately 5% around 100°C. This initial mass loss is likely due to the evaporation of residual solvents or the release of adsorbed moisture from the peptide samples. Peptides are known to be hygroscopic and readily absorb moisture from the environment. The removal of physically adsorbed water and volatile impurities at relatively low temperatures is a common phenomenon observed in TGAs of biomolecules.

At temperatures beyond the initial mass loss, the thermal behavior of *L*-PTAA differs from that of the other two peptides. *L*-PTAA begins to decompose at around 180°C. The first peak in the derivative thermogravimetric (DTG) curve for *L*-PTAA occurs at 182°C, indicating the temperature at which the maximum rate of weight loss occurs during the first decomposition step. This *T*_*d1-max*_ for L-PTAA is approximately 86°C lower than that of L-PAAT, suggesting that L-PTAA is less thermally stable than L-PAAT during the initial decomposition phase. The overall decomposition of L-PTAA proceeds through three distinct steps, as evidenced by multiple peaks in its DTG curve. This behavior may be attributed to its unique peptide sequence and structural features, which influence its thermal degradation pathway. In contrast, the thermal decomposition profiles of L-PAAT and L-PATA are quite similar. Decomposition for both peptides begins at higher temperatures compared to L-PTAA. The first temperature of maximum degradation rate (Td1-max) for both L-PAAT and L-PATA is observed around 250°C–300°C. This initial degradation step may involve the breakdown of less stable peptide bonds, side-chain decomposition, or the cleavage of weaker intermolecular interactions within the peptide structure. A second peak (Td2-max) in the DTG curves appears at higher temperatures, around 490°C–500°C for both peptides. This step likely corresponds to the decomposition of the peptide backbone, including the breaking of stronger covalent bonds and the degradation of more thermally stable structures within the peptides. The differences in thermal stability among the peptides can be attributed to variations in their amino acid sequences, side-chain functionalities, and overall molecular structures, which influence their intermolecular interactions and the energy required for decomposition.

### Antifreezing properties

#### IRI activity

**Figure 5:**
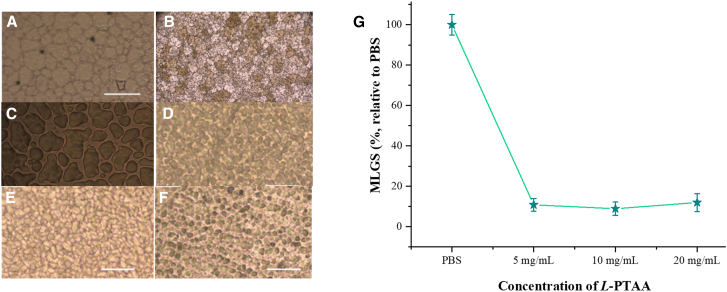
Cryomicroscope image of ice crystal annealing at −6°C for 30 min (A) Growth in PBS. (B) Growth in L-PAAT. (C) Growth in L-PATA. (D–F) Growth in L-PTAA solution (mg/mL): 5.0, 10, and 20. (G) Quantitative assessment of IRI activity of peptide at 5.0, 10, and 20 mg/mL relative to PBS solution alone. Data presented as mean (*n* = 3) largest grain size relative to PBS. Error bars represent ±SD.

IRI activity is a crucial factor for enhancing sub-zero storage and improving the preservation of biological samples. Ice recrystallization—the process where larger ice crystals grow at the expense of smaller ones during freezing and thawing cycles—can cause significant damage to cellular structures and affect the viability of preserved cells and tissues. Therefore, inhibiting this process is essential for maintaining sample integrity during cryopreservation. The IRI activities of the three synthetic AFPOs were evaluated, and the results are presented in Figures 5A–5D. Only L-PTAA demonstrated significant IRI activity; by contrast, neither L-PAAT nor L-PATA produced any appreciable inhibition of ice recrystallization. As shown in Figures 5D–5F, solutions of *L*-PTAA at both tested concentrations exhibited significantly smaller ice crystal sizes compared to the phosphate-buffered saline (PBS) control. The PBS solution, serving as the negative control, displayed larger and more irregular ice crystals, indicative of uninhibited ice recrystallization. In contrast, the presence of *L*-PTAA in the solutions resulted in a uniform distribution of smaller ice crystals, demonstrating the peptide’s remarkable IRI activity and confirming its antifreezing properties.

Quantitative analysis further supports these observations. When compared to PBS control, the mean largest grain size (MLGS) decreased rapidly upon the addition of *L*-PTAA. Specifically, at a concentration of 5.0 mg mL^−1^, the MLGS of the *L*-PTAA solution was reduced to 7% of that of the PBS blank (Figure 5G). This substantial reduction indicates that *L*-PTAA is highly effective at inhibiting ice crystal growth even at relatively low concentrations. The dramatic decrease in MLGS highlights the peptide’s ability to prevent the formation of large ice crystals, which are detrimental to cell viability during freezing processes. Interestingly, increasing the concentration of *L*-PTAA beyond 5.0 mg mL^−1^ did not result in significant additional improvements in IRI activity. This plateau in effectiveness suggests that the IRI activity of L-PTAA is not concentration dependent within the tested range. The peptide reaches its maximum inhibitory capacity at a certain concentration threshold, after which further increases do not enhance its performance. This finding is valuable as it implies that optimal antifreezing efficacy can be achieved without the need for high concentrations of the peptide, making it cost-effective and practical for large-scale applications. The non-concentration-dependent nature of *L*-PTAA’s IRI activity may be attributed to its mode of action at the molecular level. Once enough peptide molecules are present to interact with ice crystal surfaces, additional molecules may have limited access or contribute minimally to further inhibition. This saturation effect is common in antifreeze proteins and peptides, where specific interactions with ice crystal faces prevent further growth regardless of excess inhibitor presence.

#### Thermal hysteresis activity

**Figure 6:**
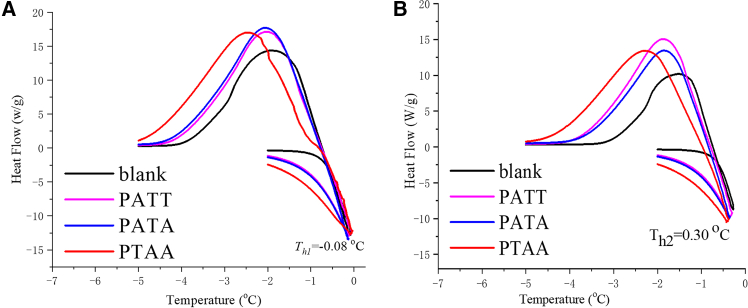
DSC curves for thermal hysteresis activity of synthetic peptides (A) Hold at −0.08°C. (B) Hold at 0.30 °C.

The freezing and melting behaviors of aqueous solutions containing the three synthesized peptides (*L*-PTAA, *L*-PAAT, and *L*-PATA) were systematically investigated by DSC. Distinct thermal events were observed in each solution. Frozen samples were heated until partial melting was achieved at specific *T*_*h*_ (−0.08°C in Figure 6A; 0.30°C in Figure 6B) and then cooled to induce recrystallization. These temperatures were selected so that only a portion of the ice was melted, thereby enabling subsequent cooling and recrystallization and allowing assessment of each peptide’s influence on ice recrystallization, a critical parameter for antifreeze efficacy. The thermal transitions occurring during heating and cooling are depicted in the DSC curves of Figure 6. Only *L*-PTAA was found to exhibit the characteristic crystallization delay upon cooling, whereas *L*-PAAT and *L-*PATA showed an exothermic onset immediately upon temperature decrease, indicating the absence of thermal hysteresis activity. Such differences are attributed to variations in monomer sequencing, which alter the spacing between threonine residues along the oligomer backbone. Interestingly, detectable thermal hysteresis is often not exhibited by antifreeze agents with moderate IRI activity. Nevertheless, a characteristic crystallization delay upon cooling was observed for L-PTAA in DSC experiments. To further clarify the protective efficacy of the synthetic AFPOs, sheep red blood cells (RBCs) were cryopreserved in their presence.

#### Cryopreservation of sheep RBCs with synthetic AFPOs

**Figure 7:**
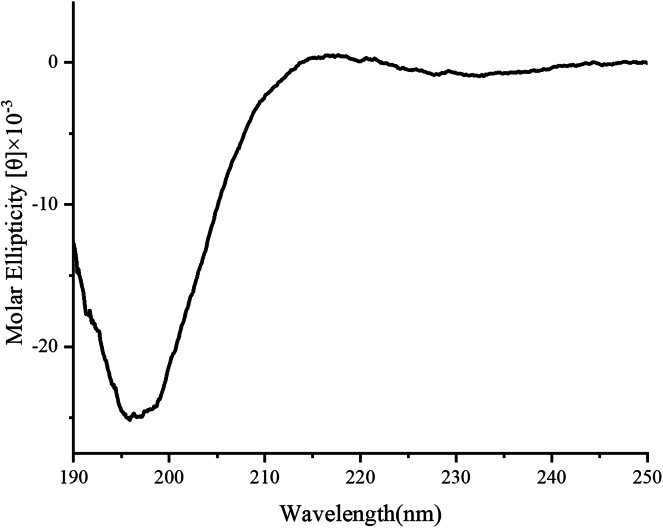
Evaluation of sheep RBC cryopreservation (A) With PBS and synthetic AFPOs (10 mg/mL). (B) With different concentration of L-PTAA. (C) Cytocompatibility of L-PTAA assessed by hemolysis following incubation with RBCs; error bars denote ±standard deviation.

Although only L-PTAA exhibited significant IRI activity, all three synthetic AFPOs were nonetheless evaluated as cryoprotectants for sheep RBCs. In freeze-thaw assays, neither L-PAAT nor L-PATA improved post-thaw RBC viability relative to L-PTAA (Figure 7A). As illustrated in Figure 7B, RBC recovery peaked at 34% when cells were frozen and thawed in the presence of 10 mg/mL L-PTAA. At both lower (5 mg/mL) and higher (15 and 20 mg/mL) AFPO concentrations, survival rates dropped significantly, underscoring a non-linear, “Goldilocks” effect that mirrors L-PTAA’s IRI profile: too little polymer cannot effectively suppress ice recrystallization, while excessive polymer may increase solution viscosity or promote osmotic stress during freezing. Importantly, long-term biocompatibility studies (Figure 7C) confirmed that incubation of sheep RBCs with 20 mg/mL L-PTAA at 4°C for 6 days did not induce measurable hemolysis, eliminating direct cytotoxicity as a cause of cell loss. Taken together, these data suggest that L-PTAA’s cryoprotection derives chiefly from its ability to modulate ice crystal growth rather than from membrane-stabilizing or metabolic effects. Further optimization of concentration and cooling rates, as well as mechanistic studies on osmotic balance, may improve cell yields toward levels required for practical blood banking applications.

#### Circular dichroism spectrum of L-PTAA

**Figure 8:**
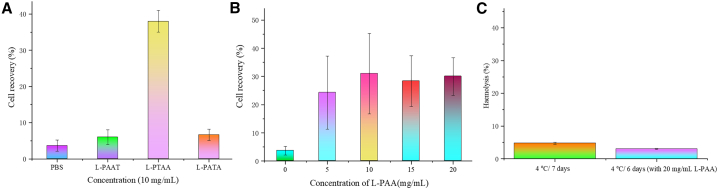
CD spectrum of L-PTAA

Circular dichroism (CD) spectroscopy was employed to analyze the secondary structure of L-PTAA in solution, to explore whether its antifreeze activity is structurally dependent. As shown in Figure 8, the CD spectrum exhibited a prominent negative peak near 197 nm, which is characteristic of intrinsically disordered (random coil) conformations.^31^ This observation suggests that L-PTAA does not adopt a well-defined secondary structure in solution. Based on this structural feature, we propose a mechanistic hypothesis that the intrinsically disordered nature of L-PTAA plays a critical role in its antifreeze activity. In contrast to folded proteins or peptides that rely on specific ice-binding motifs to interact with ice surfaces, disordered peptides like L-PTAA maintain a flexible and dynamic conformation. This structural adaptability enables the peptide to conform to the topological heterogeneity of ice crystal surfaces, thereby facilitating non-specific yet widespread molecular interactions, such as hydrogen bonding and van der Waals forces. Furthermore, the presence of hydrophilic and polar amino acid residues within the L-PTAA sequence likely allows it to interact with the quasi-liquid layer that exists at the interface of growing ice crystals. Through the formation of transient hydrogen bonds with water molecules at this interface, L-PTAA may effectively absorb onto ice surfaces and disrupt the orderly incorporation of water molecules into the crystal lattice. This interference can inhibit both ice nucleation and recrystallization, which are major contributors to cryo-induced cellular damage. Additionally, the short and unstructured backbone of L-PTAA minimizes steric hindrance, potentially allowing multiple peptide molecules to accumulate cooperatively on the ice surface, further enhancing the antifreeze effect. The peptide’s high solubility and lack of aggregation under physiological conditions also render it biocompatible, making it a promising candidate for biological cryopreservation without inducing cytotoxicity or compromising membrane integrity, issues that are often associated with conventional cryoprotectants. Collectively, these findings support the hypothesis that the intrinsically disordered structure of L-PTAA is a key determinant of its antifreeze behavior, enabling versatile surface interactions and biological safety, even in the absence of classical structural motifs.

In summary, IRI assays, DSC, and sheep RBC cryopreservation experiments collectively demonstrate that antifreeze activity is exhibited exclusively by L-PTAA, whereas its isomeric analogs L-PAAT and L-PATA remain inactive. This selectivity arises from the precise alternation of hydrophobic alanine and hydroxyl-bearing threonine residues in L-PTAA, which positions threonine at the N terminus and promotes the formation of amphipathic secondary structures. Within these structures, threonine -OH groups are regularly arrayed on one face to match the ice lattice spacing, enabling transient hydrogen bonding with the quasi-liquid layer at ice surfaces. These interactions anchor the peptide and arrest crystal growth. By contrast, shifting threonine to the C terminus in L-PAAT disrupts the periodic alignment of -OH groups with the ice-plane geometry, and in L-PATA, the hydroxyl array becomes discontinuous; in both cases, a coherent, lattice-matched hydrogen-bond network cannot be established. Furthermore, only L-PTAA’s specific alanine-threonine motif fosters dense backbone packing and organizes interfacial water into non-freezable clusters. As a result, potent ice-recrystallization inhibition and freeze-thaw protection is conferred solely by L-PTAA.

### Conclusions

An enzyme-catalyzed route to AFPOs was established using BSAP. Conventional chemical methods were replaced by this biocatalytic approach, resulting in a greener, more efficient synthesis. Reaction conditions—most notably the solvent system—were systematically optimized, yielding peptides with degrees of polymerization up to 14, as confirmed by MALDI-TOF mass spectrometry. The formation of long-chain architectures and robust peptide bonds essential for antifreeze function was further verified by ^1^H-NMR and MALDI-TOF analyses. Although identical amino acid compositions were shared by all AFPOs, measurable antifreeze activity was exhibited only by *L*-PTAA, indicating that monomer sequence—and thus detailed backbone architecture—rather than composition alone governs antifreeze performance. Excellent antifreezing properties were demonstrated by *L*-PTAA, marking it as safe, sustainable alternatives to conventional cryoprotectants. Future work will be focused on elucidating ice-inhibition mechanisms and on scaling up production processes for commercial applications.

### Limitations of the study

A primary limitation of the current work is the lack of molecular-level insight into how the AFPOs interact with water and ice to exert their antifreeze function. We have not yet carried out atomistic modeling or ice-binding simulations to reveal how the alternating alanine-threonine motif in L-PTAA organizes interfacial water into non-freezing clusters or establishes a coherent hydrogen-bond registry with the ice lattice. As a result, the precise origins of both ice-recrystallization inhibition and the observed thermal hysteresis remain speculative. Without spectroscopic or computational structural data, we cannot define the sequence and chain-length parameters that govern polymer-water interactions or explain why thermal hysteresis co-occurs with recrystallization suppression. Finally, because our products remain in the oligomeric regime, strategies to increase the degree of polymerization should be pursued in future studies to assess how chain length influences antifreeze efficacy and to optimize cryoprotective performance across a broader range of conditions.

## Resource availability

### Lead contact

Further information and requests for resources and reagents should be directed to and will be fulfilled by the lead contact, Bo Xia (11106142@zju.edu.cn).

### Materials availability

This study did not generate new unique reagents.

### Data and code availability

• This study does not report new datasets of a standardized datatype requiring public repository deposition. All data supporting the findings are included within the article and supplemental information.
• No original or custom code was generated for this study. All analyses were performed using standard commercial software packages as detailed in the STAR Methods section.
• Any additional information required to reanalyze the data reported in this paper is available from the lead contact upon request.

## Acknowledgments

This study was supported by the Research Foundation of Jiyang College of Zhejiang A&F University (RC2023S02).

## Author contributions

X.P. searched for funding and investigated the antifreezing properties; Q.W. supervised this project and reviewed the manuscript; and B.X. investigated and wrote the original manuscript.

## Declaration of interests

The authors declare no competing interests.

## STAR★Methods

### Key resources table

**Table:** 

REAGENT or RESOURCE	SOURCE	IDENTIFIER
**Chemicals, peptides, and recombinant proteins**		

Alkaline protease from *Bacillus Subtilis* (BSAP; ≥10 000 U/g)	Xuemei (Wuxi, China)	-
Novozym 435	Novonesis Ltd.	–
*Cbz-L-*alanine methyl ester	Energy Chemical Ltd.	Cat# 28819-05-8
*L-*threonine methyl ester	Energy Chemical Ltd.	Cat# 3373-59-9
*N,N*′-Dicyclohexylcarbodiimide	Energy Chemical Ltd.	Cat# 538-75-0
1-hydroxybenzotriazole	Energy Chemical Ltd.	Cat# 2592-95-2
NaOH	Energy Chemical Ltd.	Cat# 1310-73-2

**Experimental models: Cell lines**		

Sheep Red Blood Cells (20%)	Shanghai Guchen Biotechnology Co., Ltd.	–

**Software**		

Origin 2021	OriginLab Corporation.	–

### Method details

#### Synthesis of monomers

The method for methyl tripeptides synthesis was adapted from the literature (Figure 1).^22^^,^^23^^,^^24^

The procedure is described as follows.

• To a solution of N-Cbz-L-alanine methyl ester (Comp. 1, 0.1 mol) in DCM (10 mL) were added dicyclohexylcarbodiimide (0.11 mol) and 1-hydroxybenzotriazole (0.12 mol). After stirring at room temperature (R.T.) for 15 min, L-alanine methyl ester (Comp. 2, 0.1 mol) was added to the reaction mixture, and the solution was stirred overnight at R.T. Upon completion of the reaction, the mixture was concentrated under vacuum, and the residue was purified by column chromatography (petroleum ether/EtOAc = 15:1) to afford N-Cbz-Ala-Ala methyl ester (Comp. 3) in 45% yield.
• Compound 3 (0.01 mol) was dissolved in MeOH (20 mL), and a NaOH solution (2 N) was added dropwise at R.T. After 3 h, the reaction mixture was acidified with 10% HCl solution. The aqueous phase was extracted with DCM (20 mL × 3), and the combined organic layers were dried over MgSO_4_. The DCM solution was then concentrated under vacuum to afford N-Cbz-Ala-Ala (Comp. 4) in 86% yield.
• To a solution of Comp. 4 (0.1 mol) in DCM (10 mL) were added dicyclohexylcarbodiimide (0.11 mol) and 1-hydroxybenzotriazole (0.12 mol). After stirring at R.T. for 15 min, L-threonine methyl ester (Comp. 5, 0.1 mol) was added to the reaction mixture, and the solution was stirred overnight at R.T. Upon completion of the reaction, the mixture was concentrated under vacuum, and the residue was purified by column chromatography (petroleum ether/EtOAc = 15:1) to afford N-Cbz-Ala-Ala-Thr (Comp. 6) in 42% yield.
• Compound 6 was dissolved in MeOH (10 mL), and Pd/C (5% by weight) was added to the mixture. The reaction mixture was stirred at r.t. overnight. Upon completion of the reaction, the mixture was filtered, and the filtrate was concentrated under vacuum to afford the Ala-Ala-Thr monomer with a 90% yield.

The monomers L-TAA and L-ATA were synthesized according to the above general procedure.

##### *L-*Methyl alanyl-alanyl-threonine (*L-*AAT)

White solid, IR (KBr) ν: 3507, 2939, 2865, 1698, 1603, 1520, 1343 cm^−1^; ^1^H-NMR (400 MHz, D_2_O): 4.40–4.42 (d, 1H), 4.27–4.32 (m, 2H), 3.66 (s, 1H), 3.48–3.54 (m, 1H), 1.41–1.42 (d, 3H), 1.31–1.33 (d, 3H), 1.08–1.09 (d, 3H) ppm; ^13^C-NMR(400 MHz, D_2_O): 174.5, 173.4, 171.3, 67.6, 59.1, 52.8, 49.4, 48.7, 18.4, 16.4, 15.8 ppm.

##### *L-*Methyl alanyl-threonyl-alanine (*L-*ATA)

White solid, IR (KBr) ν: 3507, 2939, 2865, 1698, 1603, 1520, 1343 cm^−1^; ^1^H-NMR (400 MHz, D_2_O): 4.28–4.32 (m, 1H), 4.15–4.17 (d, 1H), 4.02–3.98 (m, 1H), 3.61 (s, 1H), 3.53–3.59 (m, 1H), 1.29–1.31 (d, 3H), 1.18–1.20 (d, 3H), 1.12–1.14 (d, 3H) ppm; ^13^C- NMR(400 MHz, D_2_O): 174.7, 173.3, 171.3, 67.1, 59.3, 52.8, 48.7, 45.6, 18.8, 17.5, 15.9 ppm.

##### *L-*Methyl threonyl -alanyl-alanine (*L-*TAA)

White solid, IR (KBr) ν: 3507, 2939, 2865, 1698, 1603, 1520, 1343 cm^−1^; ^1^H-NMR (400 MHz, D_2_O): 4.18–4.28 (m, 2H), 3.82–3.85 (m, 1H), 3.60 (s, 1H), 3.23–3.24 (d, 1H), 1.26–1.28 (d, 6H), 1.07–1.09 (d, 3H) ppm; ^13^C-NMR(400 MHz, D_2_O): 174.9, 173.7, 171.3, 70.3, 59.1, 54.2, 51.4, 50.7, 19.0, 17.9, 17.3 ppm.

#### Enzymatic polymerization

Methyl tripeptides (500 mg) and BSAP or other enzyme (20 wt % with respect to monomer, 100 mg) were added into different organic solvent. The reactions were carried out under 75°C at 200 rpm for 2–4 days and then quenched by the addition of 10 mL Methanol. The insoluble enzyme was separated by filtration, and the methanol was then evaporated under reduced pressure to obtain the crude product. The obtained polymers were dissolved in 1.0 mL methanol, and 5.0 mL ethyl acetate was added slowly. After 10 min of balance, the supernatant was discarded, and the residue was collected and retreated with this procedure three times to obtain the polymer as white powder. The molecular structure was analyzed by ^1^H- NMR and MALDI-TOF spectra. The reaction conditions, including the reaction time, enzyme sources, enzyme loading amount, and reaction temperature, were also optimized.

##### *L-*poly (methyl alanyl-alanyl-threonine) (*L-*PAAT)

White solid, ^1^H-NMR (400 MHz, D_2_O): 4.40–4.41 (d, 1H, -CH- of the chiral carbon of terminal Thr), 3.96–4.34 (m, 22H, -CH- of the chiral carbon of medial Thr or Ala), 3.66 (s, 3H, -COOCH_3_ of the end group), 3.48–3.54 (m, 1H, -CH- of the chiral carbon linked with terminal –NH_2_), 1.42–1.44 (d, 6H, -CH_3_ of Ala), 1.23–1.33 (m, 29H, -CH_3_ of Ala), 1.04–1.15 (m, 19H, -CH_3_ of Thr) ppm.

##### *L-*poly (methyl alanyl-threonine-alanyl) (*L-*PATA)

White solid, ^1^H-NMR (400 MHz, D_2_O): 3.98–4.42 (m, 23H, -CH- of the chiral carbon of Thr or Ala), 3.65 (m, 3H, -COOCH_3_ of the end group), 3.46–3.57 (m, 1H, -CH- of the chiral carbon linked with terminal –NH_2_), 1.22–1.47 (m, 39H, -CH_3_ of Ala), 1.08–1.16 (m, 21H, -CH_3_ of Thr) ppm.

##### *L-*poly (methyl threonine-alanyl-alanyl) (*L-*PTAA)

White solid, ^1^H-NMR (400 MHz, D_2_O): 3.95–4.29 (m, 16H, -CH- of the chiral carbon of Thr or Ala), 3.62 (m, 3H, -COOCH_3_ of the end group), 3.44–3.46 (d, 1H, -CH- of the chiral carbon linked with terminal –NH_2_), 1.09–1.36 (m, 38H, -CH_3_ of Ala or Thr) ppm.

#### Nuclear magnetic resonance (NMR) analysis

^1^H-NMR and ^13^C-NMR spectra were recorded with tetramethylsilane (TMS) as the internal standard using a Bruker AMX-400 MHz spectrometer (Rheinstetten, Germany). The average degree of polymerization (DP) was calculated by ^1^H-NMR spectroscopy.

#### Matrix-assisted laser desorption/ionization time-of-flight (MALDI-TOF)

MALDI-TOF spectra data were obtained using an OmniFlex MALDI-TOF mass spectrometer (Bruker Daltonics Inc.) with 1-HCCA or 1-DHB as a matrix. The matrix was dissolved in a mixture containing 0.1% trifluoroacetic acid, 70% acetonitrile, and 30% water.

#### Thermal analysis

Thermal analysis was carried out with a TA-Q500 TGA and TA Q200 Instrument, with the calorimeter under nitrogen connected to a cryostat from the same manufacturer. Thermogravimetric (TG) scans were running at temperatures ranging from 25°C to 800 °C with a heating rate of 10 °C/min.

#### IRI activity

To demonstrate IRI activity, a modified ‘splat’ assay was conducted. The cover glass is placed in Linkam BCS196 (N2(l) cooled) cryostage, cooling to −80°C. A 10 μL droplet is dropped 1.5 m onto a coverslip. Then, the system was annealed at −6°C for 30 min, temperature keeping ice by polarized light microscope in the process of recrystallization domain the size and shape of the face for “real time” *in situ* observation.^20^^,^^21^ Afterward, the wafers are imaged using an Olympus CX41 microscope equipped with a UIS-210x/0.25/N/-/FN22 lens (Olympus Ltd, Southend on sea, UK) and a Canon EOS500D SLR digital camera (Canon (UK) Ltd, Surrey, UK) through crossed polarisers. The sizes of the ten largest crystals were measured using the freely available image-processing software ImageJ and the mean largest grain size expressed as a percentage of a PBS control.

#### Thermal hysteresis activity

DSC was used to investigate the antifreezing ability of obtained peptides. In initial experiments the sample was cooled as rapidly as possible to −30°C and equilibrated at this temperature. It was then warmed to a hold temperature (*T*_*h*_, −0.08°C or 0.30°C) at 1.0°C/min and held at that temperature for 1.0°C/min. After the hold, the sample was cooled to −5.0°C and a crystallization temperature *T*_*c*_ appeared. All the *T*_*h*_ and *T*_*c*_ data were collected and the antifreezing ability of peptides was related to the differential between *T*_*h*_ and *T*_*c*_.

#### Circular dichroism (CD) determination

CD spectra of L-PTAA solutions were recorded on a CD spectrometer (JASCO-J1500). The spectra were recorded at a 0.1 nm interval from 260 to 190 nm. Experiments were performed in a rectangular cell with an optical path of 1.0 mm.

#### Cryopreservation of sheep red blood cells with synthetic AFPOs

##### Measurement of sheep RBC haemolysis and cell recovery

An initial 40 μL of the sheep RBC suspension was diluted in 400 μL of PBS and centrifuged at 10,000 rpm for 5 min at 4 °C to remove intact cells. From the resulting supernatant, 50 μL was mixed with 150 μL of PBS in a 96-well plate, and absorbance at 450 nm was recorded to quantify released hemoglobin. For the 100% hemolysis control, 500 μL of sheep RBC was combined with 500 μL of deionized water, vortexed vigorously, frozen without cryoprotectant, and thawed slowly to induce complete cell rupture. The 0% hemolysis baseline was generated by incubating 500 μL of RBCs with 500 μL of PBS at room temperature (23 °C) for 60 min without further treatment. Cell recovery was calculated by subtracting the attained hemolysis (%) from 100 (%) giving cell recovery (%).

##### Cryopreservation of RBCs with synthetic AFPOs

Sheep red blood cell suspensions (500 μL), prepared in five replicates, were combined with an equal volume of synthetic AFPO solution in PBS inside 1.8 mL cryovials and gently inverted to mix. Each vial was plunged into a dry-ice/isopropanol slush (−78 °C) for 60 s, then kept on dry ice for 20 min. Samples were thawed at room temperature (23 °C) for 30 min, and cell recovery was assessed as described above.

### Quantification and statistical analysis

Origin 2021 was used to perform the curve of TGA and DSC (Figures 4, 5, and 6). The Origin 2021 functions of first order derivative was used to perform the curve of TGA (Figure 4).
