# Protease-catalyzed enzymatic synthesis of antifreeze peptide oligomers and their cryopreservation performance

**DOI:** 10.1016/j.isci.2025.114109

**Published:** 2025-11-18

**Authors:** Xiaocheng Pan, Qi Wu, Bo Xia

## Main text

(iScience *28*, 113802; November 21, 2025)

Due to a production error during proofing, the images for Figures 7 and 8 were erroneously swapped in the original publication. This has now been corrected online. The production team apologizes for any inconvenience caused.

