# Supplementary material for: Protease-catalyzed enzymatic synthesis of antifreeze peptide oligomers and their cryopreservation performance
Source: iScience. 2025 Oct 24;28(11):113802. doi: 10.1016/j.isci.2025.113802 (PMC12651731; doi:10.1016/j.isci.2025.113802)
Supplement: Document S1. Figures S1–S7 [file mmc1.pdf]

iScience, Volume *28*

Supplemental information

Protease-catalyzed enzymatic synthesis of antifreeze peptide oligomers and their cryopreservation performance

Xiaocheng Pan, Qi Wu, and Bo Xia

Supporting Information

1. NMR spectra of monomers and polymers

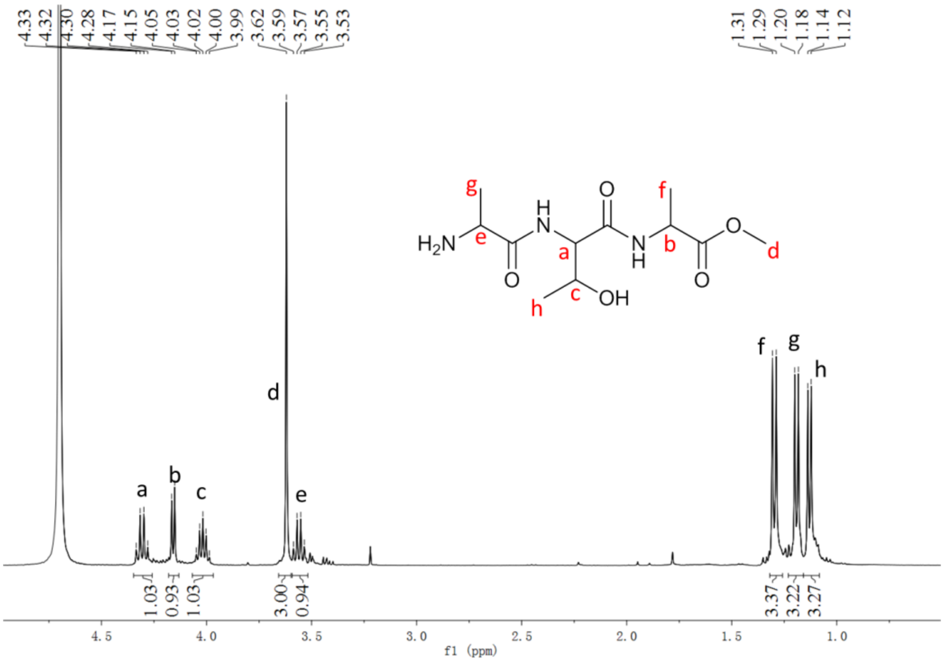

Figure S1 ^{1}H NMR of ***L*-ATA**

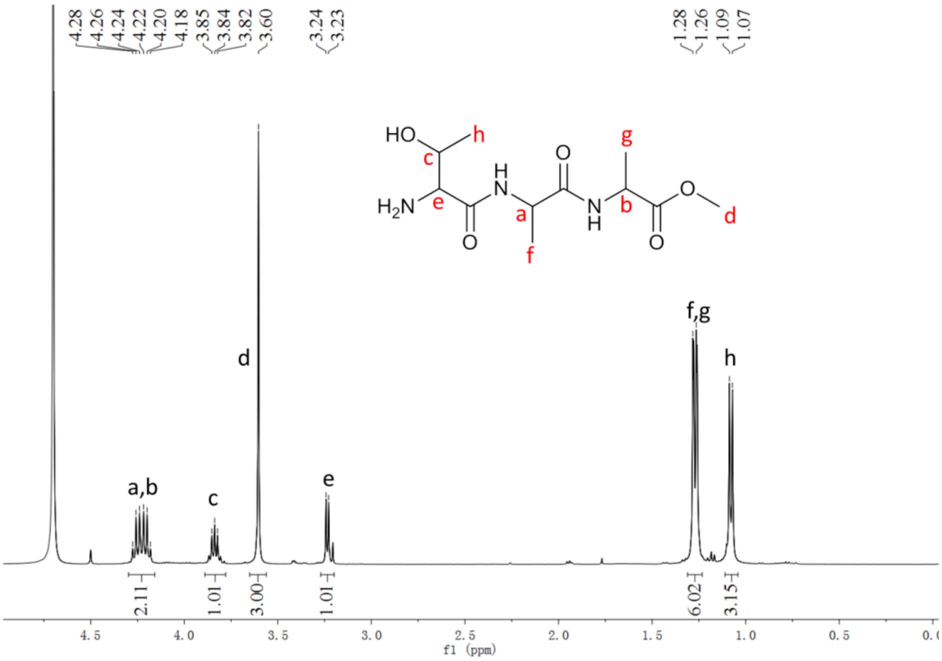

Figure S2 ^{1}H NMR of ***L*-TAA**

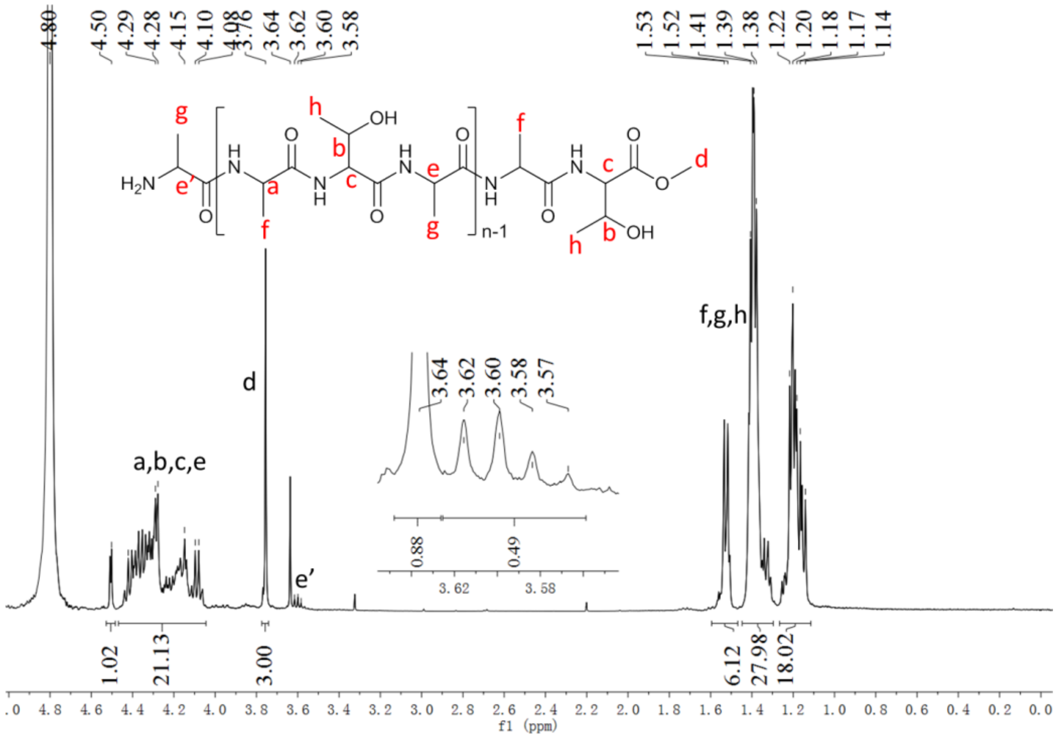

Figure S3 ^{1}H NMR of ***L*-PATA**

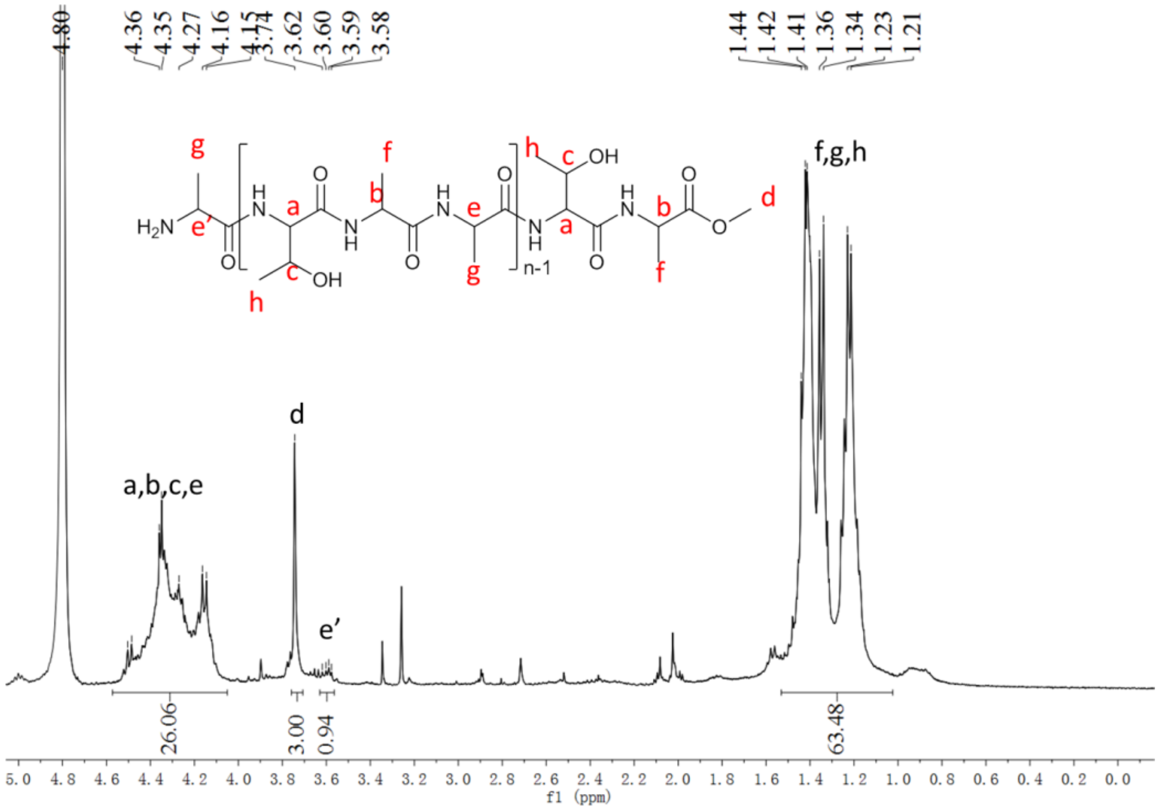

Figure S4 ^{1}H NMR of ***L*-PTAA**

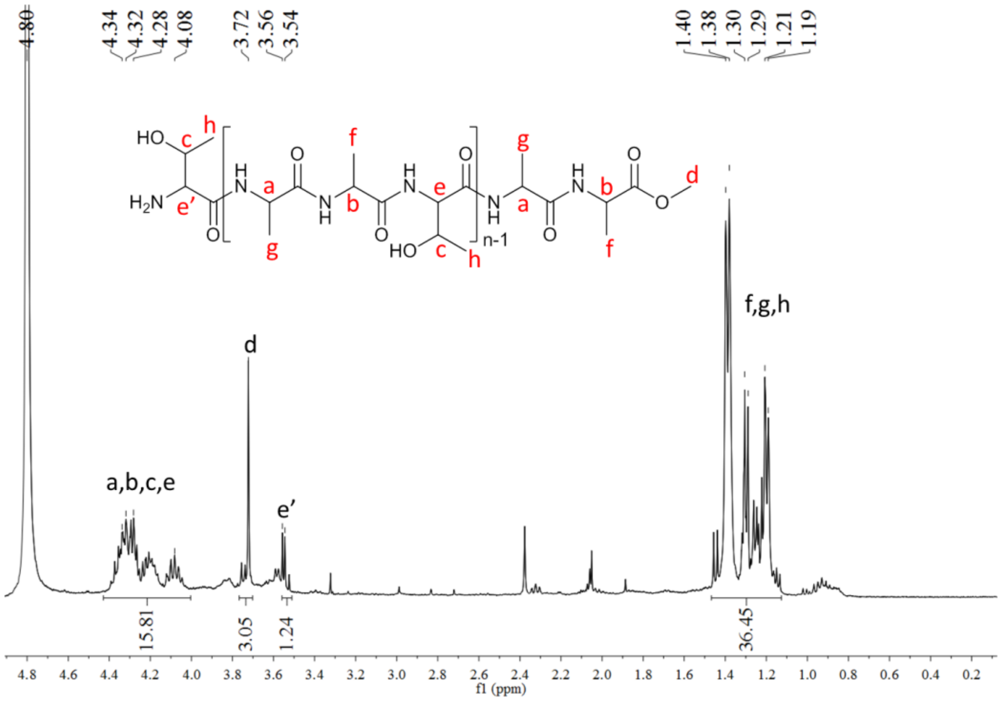

Figure S5 ^{1}H NMR of ***L*-PAAT**

2. MALDI-TOF mass spectra of polymers

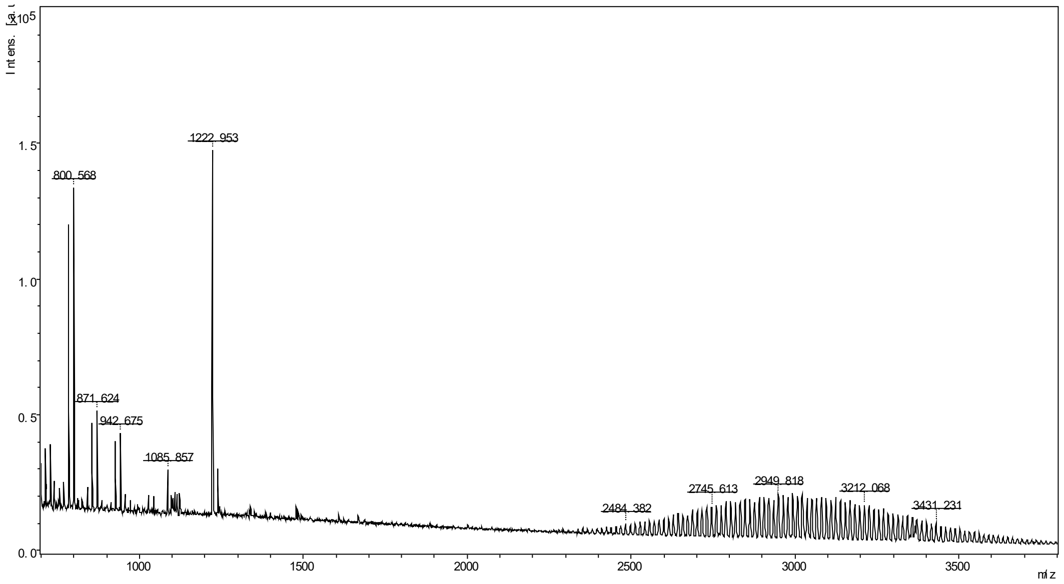

m/z	PATA	Cation	m/z	PATA	Cation
800	$(ATA)_3$	K^+	871	$A(ATA)_3$	K^+
943	$AA(ATA)_3$	K^+	1223	$TA(ATA)_4$	K^+
2484	$(ATA)_{10}$	Na^+	2745	$(ATA)_{11}$	K^+
2949	$(ATA)_{12}$	H^+	3212	$(ATA)_{13}$	Na^+
3431	$(ATA)_{14}$	H^+			

Figure S6 MALDI-TOF mass spectrum of ***L*-PATA**

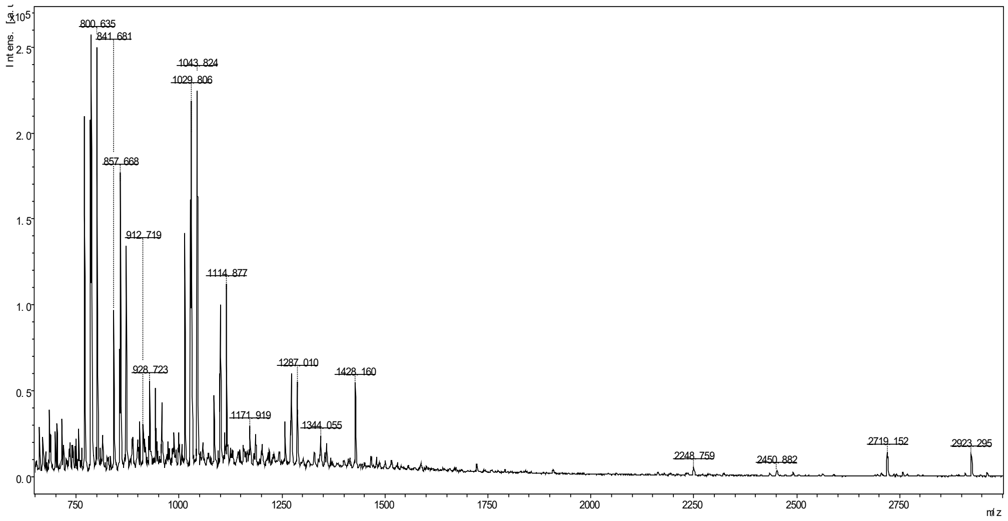

m/z	P TAA	Cation	m/z	P TAA	Cation
800	$(TAA)_3$	K^+	871	A $(TAA)_3$	K^+
912	$AA(TAA)_3$	Na^+	928	$AA(TAA)_3$	K^+
1029	$(TAA)_4$	Na^+	1043	$(TAA)_4$	K^+
1114	A(TAA)4	K^+	1287	$(TAA)_5$	K^+
1428	$AA(TAA)_5$	K^+	2248	$(TAA)_9$	K^+
2719	$(TAA)_{11}$	NH_4^+			

Figure S7 MALDI-TOF mass spectrum of ***L*-PTAA**
